# Polyamines in aging and disease

**DOI:** 10.18632/aging.100361

**Published:** 2011-08-21

**Authors:** Nadège Minois, Didac Carmona-Gutierrez, Frank Madeo

**Affiliations:** ^1^ University of St Andrews, School of Biology, Bute Building, St Andrews, Fife, KY16 9TS, UK; ^2^ Institute of Molecular Biosciences, University of Graz, 8010 Graz, Austria

**Keywords:** Polyamines, Aging, Age-related diseases, Cancer, Autophagy, Acetylation

## Abstract

Polyamines are polycations that interact with negatively charged molecules such as DNA, RNA and proteins. They play multiple roles in cell growth, survival and proliferation. Changes in polyamine levels have been associated with aging and diseases. Their levels decline continuously with age and polyamine (spermidine or high-polyamine diet) supplementation increases life span in model organisms. Polyamines have also been involved in stress resistance. On the other hand, polyamines are increased in cancer cells and are a target for potential chemotherapeutic agents. In this review, we bring together these various results and draw a picture of the state of our knowledge on the roles of polyamines in aging, stress and diseases.

## INTRODUCTION

**Figure 1:**
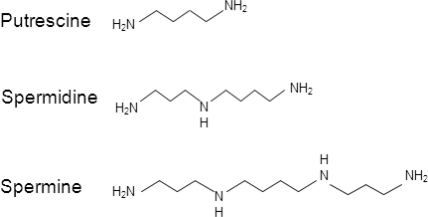
Putrescine, spermidine and spermine chemical structure

Polyamines have been known for a long-time as the first one, spermine, was discovered over 330 years ago by microscopic observation of human semen [reviewed in 1]. They have since been found in all eukaryotes and most prokaryotes. Polyamines are polycations (Figure 1) and thus one of their main features is to interact with negatively charged molecules, such as DNA, RNA or proteins. Given their promiscuity in binding other molecules, they are involved in many functions, mostly linked with cell growth, survival and proliferation. Three polyamines, putrescine, spermidine and spermine, are part of the very tightly regulated polyamine metabolic pathway. Polyamines are the subject of intensive research in order to elucidate their functions and involvement in physiology. Polyamines are important players in plant growth, stress and disease resistance [2], but they are also involved in diseases [3], for example Alzheimer's or infectious diseases. The main research area for the involvement of polyamines in diseases is cancer, as high levels of polyamines are observed in cancer cells [3]. More recently, we have shown a causative role for polyamines in longevity [4].

This review will give an update on the field of polyamine research and begin with their metabolism and transport. Then we will discuss their involvement in aging, stress, diseases with a special section on cancer, and what is known of their mechanisms of action. We argue that only by gathering information from all the various disciplines studying polyamines can we draw an accurate picture of their effects and mechanisms of action.

### Polyamine metabolism and transport

**Figure 2:**
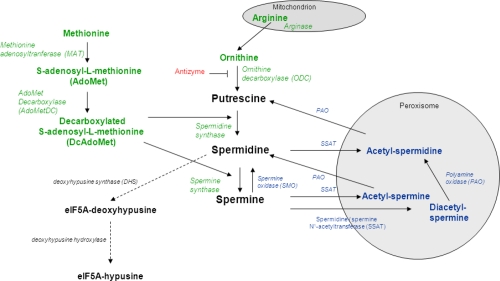
Polyamine metabolism. Green: biosynthesis; blue: catabolism; red: inhibitory protein; black: eIF5A synthesis from spermidine

The regulation of polyamine levels is achieved by a combination of synthesis, catabolism and transport. Below is a summary of these different processes. Polyamine metabolism is summarized in Figure 2. For further details, several excellent reviews and papers on this topic have been published and the reader is referred to them and the references therein [5-8].

#### Polyamine synthesis

Three main sources for polyamines exist in organisms: Food intake, cellular synthesis, microbial synthesis in the gut.

Polyamines are synthesized from the amino acids arginine, ornithine and methionine. The first step in the pathway is the production of ornithine from arginine by the mitochondrial enzyme arginase. Ornithine is then decarboxylated by ornithine decarboxylase (ODC) to produce putrescine. ODC expression is tightly regulated from transcription to post-translational modifications. ODC antizyme directly inhibits ODC activity and is also responsible for facilitating ODC degradation by targeting it to the 26S proteasome. In parallel to putrescine production, L-methionine is converted into S-adenosyl-L-methionine (AdoMet), which is then decarboxylated by AdoMet decarboxylase (AdoMetDC) to produce decarboxylated AdoMet (DcAdoMet). DcAdoMet is then used as an aminopropyl group donor either to putrescine by spermidine synthase to produce spermidine, or to spermidine to produce spermine by spermine synthase.

#### Polyamine catabolism

The higher polyamines spermidine and spermine can be converted back to putrescine (Figure 2). The rate-limiting enzyme of polyamine catabolism is the cytosolic spermidine/spermine N^1^-acetyltransferase (SSAT). SSAT acetylates both spermine and spermidine. Acetylated spermine and spermidine then move into the peroxisome where they are oxidized by polyamine oxidase (PAO). By-products of this oxidation include hydrogen peroxide (H_2_O_2_) and acetaminopropanal. SSAT is absolutely necessary for the formation of putrescine from spermidine. Spermine can also be back-converted into spermidine by spermine oxidase (SMO) in the cytoplasm. In contrast with PAO, the preferred substrate of SMO is spermine itself and not its acetylated derivative, acetylspermine.

#### Polyamine transport

Polyamine transport plays an essential role in polyamine levels regulation. Polyamine transport is well characterized in the bacterium *Escherichia coli* and the yeast *Saccharomyces cerevisiae*. Polyamine transport complexes have also been studied in plants.

*E. coli* has two polyamine uptake systems belonging to the ABC transporters family. One system is a spermidine-preferential system and the second one a putrescine-specific system. Each system consists of 4 transporters: PotA to D for spermidine transport and PotF to I for putrescine transport. If any of the spermidine transporters is missing, spermidine uptake is abolished. There are also two exporters (PotE and CadB), uptaking polyamines at neutral pH and excreting them at acidic pH. Finally, a spermidine excretion protein, MdtII was recently identified [9].

*S. cerevisiae* polyamine transport is energy-dependent and regulated mainly by phosphorylation and dephosphorylation. The proteins DUR3 and SAM3, and to a lesser extent GAP1 and AGP2, are responsible for polyamine uptake across the plasma membrane. Putrescine can be taken into the vacuole by the 4-aminobutyric acid transporter UGA4. TPO1 to 4 excrete polyamines at acidic pH (at which yeast cells usually grow) but uptake them into the yeast cell at pH 8. A polyamine preferential excretion protein, TPO5, has been identified on Golgi and post-Golgi vesicles. Recently, Teixeira et al. [10] showed that the gene QDR3, coding for a plasma membrane drug: H^+^ antiporter, is involved in resistance to spermine and spermidine, but not putrescine in yeast. *Δqdr3* yeast cells grew less when plated on food containing spermidine or spermine at high concentrations. They accumulated more spermidine, suggesting it is likely involved in polyamine excretion.

In mammals, the TATA-binding associated factor 7 (TAF7) rescues the lack of polyamine transport in methylglyoxal bis (guanylhydrazone) Resistant Chinese hamster ovary cells [11]. Antizyme, the protein responsible for ODC inhibition and degradation also enhanced polyamines and acetylpolyamines excretion. Finally, a diamine transporter has been identified in colon epithelial cells, which could be responsible for putrescine as well as acetylated polyamines excretion [12]. Despite these studies and substantial research in this area, no polyamine transporter has been identified in mammals. Alternatively, it is thought that polyamine uptake in mammals could be performed by endocytosis. Still, a set of characteristics that any polyamine transporter should fit has been defined and should help in the identification of polyamine transporters in mammals [13]. We have carried out a search for human homologues for the known polyamine transporters in *E. coli* and *S. cerevisiae* (Table 1 and supplementary table). It would now be interesting to study the hits we found and assess whether some of them can really transport polyamines.

**Table 1:** Number of human homologues for the Escherichia coli and Saccharomyces cerevisiae polyamine transporters The amino acid sequences were retrieved from the NCBI protein database or the SGD database for polyamine transporters of *Escherichia coli* and *Saccharomyces cerevisiae* and subjected to a standard blast search in NCBI (algorithm blastp). The chosen query database was “RefSeq Protein”. The search was restricted to *Homo sapiens*. For CadB (*E. coli*) two possible sequences were retrieved (147 and 444 amino acids) and both were used in our search. The shorter protein is part of the longer one. For MdtII no protein but rather a chain A and a chain B of the Mdt protein were found. Both chains were used in our search. For all transporters, only the homologues with an E value below 10^-4^ were included. Applying these parameters, the list of proteins yielded in this search was big. The results can be subdivided into 3 groups: (i) those proteins without any apparent homolog; (ii) proteins with 1-6 homologs and (iii) proteins with a long list of homologs. While the result for group (i) is clear, the results for group (ii) are all at the edge of the applied threshold and none of them is an overwhelming hit. As for group (iii) the long list might be due to the homology of a conserved functional domain and to the inclusion of all isoforms of a specific hit (see e.g. homologues for PotA in *E. coli*). The full list of homologues can be found in the Supplementary Table.

Transporters	Number of human homologues
*Saccharomyces cerevisiae*	
QDR3, TPO4, TPO5	1
TPO2, SAM3	2
TPO3	3
AGP2, DUR3	5
TPO1, GAP1	6
UGT4	19
*Escherichia coli*	
PotB, PotC, PotH, PotI	0
PotD, PotF	1
CadB	10
PotE	12
PotA, PotG	75+

### Polyamines and aging

Polyamine levels decrease with age in many organisms [reviewed in 14], albeit polyamine- and tissue-specifically. For instance, Nishimura et al. [15] measured polyamine levels in 14 different tissues in 3, 10 and 26 week-old female mice and found that spermidine levels decreased in 11 out of the 14 tissues. In contrast, spermine decreased only in skin, heart and muscles. Putrescine levels were very low in all tissues at all ages. Vivó et al. [16] reported a negative correlation between spermidine content and age in several areas of the basal ganglia in human brains and a similar trend for spermine. More varied age-related changes have been observed when more brain areas were studied in rats [17]. We reported a decrease with age of spermidine content in yeast [4]. The concentrations of specific polyamines and molecular conjugates varied with stages of growth (seedlings, saplings and mature trees) in *Pinus radiata* [18]. The same authors reported that the total polyamine level increased but no difference in free polyamines was observed in *Prunus persica*. Despite these correlative changes in polyamines with age, it is only recently that the causative involvement of polyamines in the aging process has been investigated.

The study of the effects of polyamines on aging seems to be more advanced in plant biology, where they have been identified as “juvenility” factors for some time. For instance, Serafini-Fracassini et al. [19] showed that spermine treatment delayed senescence in excised flowers of *Nicotiana tabacum*. After 48 hours, 70% of the flowers were still at the stage at which they had been cut (anthesis or peak bloom) whereas in the controls, 50% were at an early senescence stage and the other 50% senescent. Spermine delayed DNA degradation and preserved chlorophyll content. Spermidine and putrescine also delayed senescence of excised flowers, but to a lesser extent than spermine. More recently, the same group [20] studied senescence in *Lactuca sativa*. In cut leaves, spermine postponed chlorophyll loss, as observed in *N. tabacum*, probably via chlorophyll stabilization by a higher transglutaminase activity. In intact plants, the same preservation of chlorophyll and higher transglutaminase activity were observed after spermine spraying, especially in senescent plants. In contrast, the treatment did not affect the protein content decrease observed with age.

Transgenic animal models, particularly rodents, have been designed to modulate the activity of the polyamine pathway. However, these models have rarely been used in the context of studying aging. Suppola et al. [21] reported the creation of a mouse model overexpressing both ODC and SSAT under the methallothionein I promoter. These mice accumulated high levels of putrescine and exhibited a depletion of spermine and spermidine. They showed no overt organ-specific histopathological changes, but permanently lost their hair at 8 to 9 weeks of age. This hair loss was already observed in single transgenics for SSAT overexpression, which also exhibited extensive wrinkling upon aging. Finally, the double transgenic mice were very short-lived. Cerrada-Gimenez et al. [22] also reported a decreased life span in mice overexpressing SSAT. They noticed that in these mice, p53 expression in the liver was increased and that the SSAT overexpressing mice exhibited similar aging phenotypes to mice with activated p53 expression. However, it is difficult to really know if such phenotypes reflect an acceleration of the aging process or whether they reflect a general disturbance of the organism physiology leading to general weakness.

Another strategy to study the effects of polyamines in aging is an exogenous administration to organisms. When polyamines are provided with food or water, their endogenous levels increase. For instance, Soda et al. [23] observed an increase in spermine after 26 weeks in mice and after two months in humans under a high-polyamine diet. We also reported [4] that providing spermidine in food or water increased its endogenous levels in yeast, flies, and mouse liver. This is thus a promising strategy, particularly valuable in the context of extending polyamine use to humans. Using such an experimental approach to modulate polyamine levels, Soda et al. [24] fed male mice a low, normal or high-polyamine chow. They showed that mortality in mice fed a high-polyamine chow was lower in the first 88 weeks. Unfortunately, the mice were sacrificed at 88 weeks of age, precluding the gathering of mortality data after that age. The authors also reported a lower incidence of age-related kidney glomerular atrophy kept on high-polyamine diet. Finally, they observed that old mice on high-polyamine chow kept a thicker coat with age and appeared more active. However, these last two observations were only qualitative as coat thickness and activity were not measured. The lower mortality in mice fed a high-polyamine chow was not due to a dietary restriction effect as these mice ate more than the other two groups and exhibited similar body mass measured between 11 and 55 weeks of age. These results are promising and further studies in rodents are urgently required.

We have recently published a study following the consequences of external spermidine administration in various model organisms, including yeast, worms, flies, mice and human cells [4]. Spermidine increased chronological life span in wild-type yeast as well as remaining replicative life span in old yeast cells. In contrast, a *Δspe1* yeast mutant unable to synthesize polyamines was short-lived. This decreased life span was rescued by spermidine as well as by putrescine addition. We also showed that spermidine supplementation increased life span in the nematode worm *Caenorhabditis elegans* by 15% and in the fly *Drosophila melanogaster* by up to 30%. At the cellular level, spermidine increased survival of human peripheral blood mononuclear cells after 2 days from 15% in the controls to 50% by preventing death from necrosis. These results strongly suggest that spermidine could represent a new preventive agent in our fight against aging. However, so far research has mainly focused on the effect of spermidine on life span and now it is important to study its effect, as well as the potential effects of other polyamines, on aging *per se* and quality of life (healthspan).

### Polyamines and stress

Organisms are regularly exposed to stress and the ability to resist stress is a part of survival strategies. Various studies showed that polyamines had important roles in and generally correlate with stress resistance. Again, plant biology has been at the forefront of this research area. Readers are particularly refereed to a recently published excellent review on the subject [2]. Polyamines are particularly important for adaptation and resistance to cold stress [reviewed in 25] and polyamine levels increase in plants during abiotic stress such as salinity, extreme temperature, paraquat or heavy metals. This regulation of polyamines under stress is achieved by differential expression of polyamine biosynthesis enzymes, such as arginine decarboxylase, spermidine and spermine synthase or AdoMetDC. Exogenous application of polyamines led to, in varying degrees, preserved membrane integrity and lower growth inhibition during stress, reduced accumulation of ROS and increased activity of antioxidant enzymes such as catalase. In contrast, polyamine synthesis inhibitors triggered decreased stress resistance, a phenotype counteracted by the simultaneous treatment with polyamines. Mutants in arginine decarboxylase or spermine synthase are sensitive to stress. Using another approach to study the role of polyamines in stress resistance, many groups have engineered plants to overexpress polyamine biosynthesis genes, especially arginine and ornithine decarboxylases and AdoMetDC. Many studies are described in [2] and only a few, published later are reported here. Mohapatra et al. [26] studied aluminum stress in interaction with calcium in poplar cell cultures with normal or high levels of putrescine. Reducing or increasing the amount of calcium had no effect on growth in the control cells except when drastically reduced, but respectively decreased and increased growth in cells with high levels of putrescine. The addition of aluminum with normal or low calcium again had no effect in the control cells. In contrast, the cells with high putrescine content exhibited a better growth. These latter had a lower mitochondrial activity than control cells, but a higher mitochondrial activity after 48 hours of exposure to aluminum. Taken together, these results suggest that in these cells, a high putrescine content is a disadvantage in a low-calcium environment but an advantage in the presence of aluminum. Wang et al. [27] cloned the arginine decarboxylase gene from trifoliate orange and carried out its functional study in transgenic *Arabidopsis*. The transgenic plants had higher levels of putrescine but not of spermidine and spermine. They displayed a lower stomatal density, longer roots, larger cell size and higher relative water content than the controls both in normal conditions and under stress. They had similar germination to the controls in normal conditions and germinated better under osmotic stress. During dehydration, the transgenic plants lost less water, had a better electrolyte leakage and leaf turgor. Upon sustained dehydration, they exhibited better growth, survival and electrolyte leakage. The transgenic plants were also more resistant to cold. This higher stress resistance might be due to lower accumulation of H_2_O_2_, O_2_^−^ and lipid peroxidation. Finally, *A. thaliana* overexpressing spermine synthase or wild-type exogenously supplied with spermine were shown to be more resistant to infection with *Pseudomonas viridiflava*. In contrast, mutants in spermine synthase with low levels of spermine were less resistant to the same infection [28].

In model organisms, the effect of polyamines on stress resistance has not been so widely studied, but as in plants, has shown various results. SSAT overexpressing mice exhibited an increased production of H_2_O_2_ from polyamine degradation, a higher carbonyl content, and a decreased expression of superoxide dismutase (SOD), catalase and CYP450 2E1, suggesting these mice may be more prone to stress [22]. However, these mice were more resistant to thioacetamide or carbon tetrachloride, because these compounds need to be metabolized before becoming toxic, and were not metabolized to the same degree in the transgenic mice. Kaasinen et al. [29] had earlier shown that SSAT overexpressing mice exhibited decreased spontaneous activity, climbing and wheel running behavior. The transgenic mice were more phlegmatic and less aggressive. They had increased levels of ACTH and corticosterone, and decreased levels of TSH and T4, again suggesting they may be more prone to the effects of some stress.

Putrescine, spermidine and spermine decreased survival under hypoxia in *D. melanogaster* [30]. In contrast, we have shown [4] that spermidine treatment increased resistance to heat and H_2_O_2_ in yeast. It also decreased age-related oxidative stress in mice as measured by higher levels of free thiol groups. Markers of oxidative stress were also reduced in yeast. From these studies, we can conclude that polyamines are involved in stress resistance but in a much more complex way than just allowing a general higher resistance to stress when present at higher levels.

### Polyamines and diseases

Many diseases are associated with cellular dysfunction, such as aggregation of insoluble compounds, uncontrolled cell death, and anarchic cell proliferation as in cancers. As regulators of cell growth and death, it is likely that polyamines may affect the severity and process of diseases. Polyamine levels increase in many diseases [see Table 2 in 3]. As polyamines also regulate growth in pathogens, they may have an impact on infectious and parasitic diseases as well. However, as for aging and stress resistance, polyamines have different effects in different models and on different diseases. We will review here some of the results, focusing on age-related diseases and infection. The relation between cancer and polyamines will be reviewed in the next section.

Vivó et al. [16] reported negative correlations between age and spermidine in several areas of the basal ganglia in human brain, but found no difference between controls and Parkinson's disease, Huntington's disease and progressive supranuclear palsy patient's brains. More than 90% of circulating spermidine and over 70% of spermine are associated with red blood cells. Gomes-Trolin et al. [31] observed that in red blood cells, putrescine levels decreased in Parkinson's disease and amyotrophic lateral sclerosis patients. In contrast, spermidine and spermine increased in both sets of patients. There was no correlation between the levels measured and the severity of the disease. Earlier, Yatin et al. [32] suggested a possible involvement of polyamine metabolism in Alzheimer's disease. They incubated rat embryonic hippocampal neuronal cultures with Aβ(1-42) or Aβ(25-35) peptides and/or vitamin E. They observed that both peptides induced cell death and that this was largely prevented by the addition of vitamin E. They also reported an increased of polyamine uptake and ODC activity, again largely prevented by vitamin E, except for ODC activity with Aβ(25-35) peptides that was still significantly higher. Based on these results, the authors suggested that the changes observed were a response of polyamine metabolism to peptide-associated free radical damage. However, whether these changes reflected a homeostatic defense mechanism or played a role in neurotoxicity is still a matter of debate. *In vitro* studies have shown that polyamines can alter aggregation of insoluble compounds associated with disease. For instance, Antony et al. [33] observed that all three polyamines increased α-synuclein aggregation (that leads to the formation of Lewy bodies in Parkinson's disease) in solution. This was also observed more recently by Grabenauer et al. [34], who reported that spermine increased wild-type and mutants α-synuclein aggregation in solution by inducing a collapsed conformation. However, such results are not surprising since polyamines will interact and bind with such molecules, stabilizing them into aggregates when in solutions. Studies looking at the effect of polyamines on the aggregation of such compounds *in vivo* are needed. Recently, Lewandowski et al. [35] investigated the involvement of polyamines in Parkinson's disease. They found that spermine was more toxic to yeast expressing wild-type or mutant α-synuclein. Overexpression of a polyamine transporter, Tpo4, increased α-synuclein toxicity. They also observed in transgenic mice expressing wild-type human α-synuclein that neuronal accumulation of α-synuclein in the substantia nigra was increased by Berenil (that decreases SSAT activity, thus polyamine catabolism) and decreased by DENSPM (that increases SSAT activity). However, as with many similar studies, the authors were cautious in pointing out that this type of correlative observation cannot establish a pathogenic link with the disease. At the current stage of research, it cannot be ruled out that the changes observed in polyamine levels and the effects of polyamines on disease may actually be a compensatory, protective mechanism. Furthermore, different polyamines have different effects on disease. For instance, spermine exacerbated ischemic neuronal injury in rodent models of ischemia [36]. This effect of spermine was dependent on functional acid-sensing ion channels (ASICs), especially ASIC1a. However, no such damaging effect was observed with putrescine or spermidine. Spermine also increased neuronal damage in culture rat hippocampal neurons induced by oxygen and glucose deprivation. DFMO, an ODC inhibitor, attenuated neuronal damage in these cells. In contrast, polyamines can also be neuroprotective. In a *Xenopus* tadpole model of epilepsy, Bell et al. [37] observed that tadpoles primed with a first seizure induced by pentylenetetrazole (PTZ), a convulsant, exhibited a delayed onset of seizure upon a second exposure to PTZ as long as the period between both exposures was not too short (allowing recovery from first seizure). If the tadpoles were previously given DFMO, an ODC inhibitor, onset time decreased, showing a harmful effect of polyamine depletion. If the synthesis of spermidine and spermine (but not putrescine) was blocked, the primed tadpoles still had a beneficial effect of the priming, showing that the beneficial effect of priming is triggered by putrescine and not the higher polyamines. If tadpoles were incubated with putrescine before exposure to PTZ, again the seizure onset time was delayed. No such effect was observed when incubated with spermine, confirming the results using inhibitors of polyamine synthesis. They showed that neuroprotection was achieved by the conversion of putrescine into GABA, as a diamine oxidase (enzyme involved in the conversion of putrescine into GABA) inhibitor decreased seizure onset time. In an excellent review of transgenic rodents for genes of polyamine metabolism, Jänne et al. [6] reported that accumulation of putrescine in the brain can be neuroprotective too. Transgenic mice and rats overexpressing ODC and thus exhibiting high levels of putrescine, particularly in the brain and testis, showed an elevated seizure threshold, and in the transgenic rats, ischemia reperfusion damage developed more slowly with smaller infarct volumes.

Polyamines are also important in diseases such as pancreatitis. The pancreas is the organ where the highest levels of spermidine are observed in mammals. Transgenic rats overexpressing SSAT exhibited a depletion of spermidine and spermine and developed pancreatitis. Furthermore, these rats failed to initiate liver regeneration after partial hepatectomy. Liver regeneration could only begin once the spermidine levels were restored because of ODC activation. Supporting the involvement of polyamines in liver regeneration, Jung et al. [38] showed that methionine, ornithine, AdoMet, putrescine and spermidine levels were quickly upregulated in rats subjected to partial hepatectomy until the original liver weight was reached. Spermine levels were decreased because of the increased use of DcAdoMet for spermidine synthesis.

Many diseases are associated with inflammation and polyamines have been involved in inflammatory responses. Polyamine levels generally increase with inflammation. However, whether they are pro- or anti-inflammatory is still unclear. Recently, Puntambekar et al. [39] studied the dependence on polyamines of inflammation triggered by lipopolysaccharides (LPS). In microglia in culture, the treatment with LPS+/− IFNγ increased ODC, SSAT and antizyme activities, both synthesis and catabolism of polyamines. Intracerebral injection only increased ODC activity. This higher activity led to the influx of pro-inflammatory macrophages into the CNS. This recruitment was mediated by the induction of CCL2, a macrophage chemoattractant. Co-injection of DFMO, an ODC inhibitor, prevented CCL2 expression by LPS. Putrescine and spermine induced the accumulation of TNF (tumor necrosis factor) and CCL2 in mixed glial cultures. Spermidine did not have such an effect. The authors concluded that ODC expression was an early response to inflammation and that the increased polyamine levels resulting from ODC activation could lead to pro- or anti-inflammatory roles depending on the microenvironment. The potential anti-inflammatory role of polyamines, which also lead to the production of nitric oxide, has led Soda [40] to hypothesize that polyamine uptake may help with cardiovascular diseases. Recently, spermidine was shown to be beneficial against two age-related diseases: cataract formation and multiple sclerosis. Lentini et al. [41] demonstrated that exogenous spermidine addition in the medium delayed the progression of eye lens opacification in an *in vitro* cataract model. This was achieved by interfering with transglutaminase activity. Finally, a spermidine-treated mouse model for multiple sclerosis (myelin oligodendrocyte glycoprotein-induced experimental autoimmune encephalomyelitis mice) exhibited improved demyelination and axon survival in spinal cord and optic nerve, improved visual functions, and reduced H_2_O_2_-induced apoptosis in retinal ganglion cells [42]. To conclude, polyamines have a complex relationship with diseases. They may be harmful, neutral or beneficial, depending on the specific polyamine and disease. However, it seems that spermidine showed the most positive effects, which would be in line with its beneficial effects reported on life span and stress.

Cell proliferation is an important part of infection, whether it is multiplication of the pathogen into the host or the host mounting an immune response. By controlling cell growth and proliferation, polyamines may thus affect the outcome of infectious and parasitic diseases. Again, there is a fine balance between beneficial and deleterious effects as polyamines may increase or decrease the fitness of both pathogen and host. A few recent studies will exemplify this complexity. Nishimura et al. [43] reported that rats fed a polyamine-deficient chow survived longer an infection by *Trypanosoma brucei brucei* and were less anemic. This was not achieved by a reduction in the number of parasites, but probably by changes in cytokines and nitric oxide production, which might lessen the symptoms of infection but not the parasite proliferation. In contrast, the authors reported that a polyamine-deficient diet restrained the proliferation of *Trypanosoma brucei gambiense*. Reyes-Becerril et al. [44] studied the effect of putrescine, spermidine and spermine on seabream head-kidney leucocytes. The polyamines had no effect on peroxidase content. Putrescine increased respiratory burst activity after 4 hours and spermidine decreased it. Putrescine also increased the percentage of phagocytic cells after 2 and 4 hours but not their phagocytic activity. Finally, several genes important for the immune response were transcriptionally up-regulated by polyamines, although in a polyamine- and incubation time- specific manner. Taken together, these results suggest that polyamines can help develop an appropriate adaptive immune response.

In plants too, polyamines have been shown to play a role in diseases [reviewed in 2]. Spermine elicits a defense response to fungal and viral pathogens. *Nicotiana tabacum* overexpressing a gene responsive to spermine had an increased resistance to tobacco mosaic virus [45]. However, although polyamines can be beneficial against infection, they can also be used by the pathogen to help proliferation.

Pneumococci have a membrane polyamine transporter and some polyamine synthesis enzymes. Shah et al. [46] showed that mutant strains with deficient polyamine transport or synthesis were attenuated. They were less able to colonize the nasopharynx and cleared more rapidly. Mice infected with mutant strains, except the mutant for lysine decarboxylase, survived the infection longer than when infected with a control strain. However, the mutants showed no defect in growth, survival to paraquat or low pH and no difference in protein expression pattern.

### The special case of cancer

Cancer and proliferative cells exhibit high levels of polyamines [see Table 3 in 3] and this is thought to be a feature by which cancer cells maintain their proliferative capacity. However, the exact role of polyamines in cancer is still unclear. Forced induction of ODC activity in normal cells did not trigger oncogenic transformation [e.g., 47] although some authors [e.g., 48] reported opposite results using the same cells but different transfection vectors and ODC gene (murine *versus* human). At the level of the whole organism, Alhonen et al. [49] could not observe a higher level of spontaneous tumor incidence in transgenic mice overexpressing human ODC under its own promoter. Soda et al. [24] reported no increase in oncogenic transformation in healthy mice fed a high polyamine diet. Given the lack of conclusive evidence that polyamines by themselves can induce tumorigenesis, studies are carried out either in model organisms tumor-prone or after induced tumorigenesis. A strategy to study the involvement of polyamines in cancer has been to use polyamine biosynthesis pathway inhibitors or polyamine analogues. For instance, Bernacki et al. [50] used DENSPM, a spermine analogue, in cell culture or in mice to study tumor development. The proliferative activity of six different solid tumor cell lines decreased after the treatment. DENSPM also delayed tumor growth *in vivo* but led to weight loss and toxicity at high doses. DENSPM did not alter ODC and AdoMetDC activities but depleted the polyamine pool, especially spermidine and spermine. More recently, Kramer et al. [51] studied the effects of two inhibitors, DMFO (ODC inhibitor) and MDL-73811 (AdoMetDC inhibitor) on cell cycle arrest. Each inhibitor triggered a slight decrease in cell growth in MALME-3M cells. In contrast, the combination of both inhibitors led to a rapid cell cycle arrest, with an accumulation of cells in G1 and a reduction in S phase. This cell cycle arrest was reversible if not prolonged. They noted a strong p21 induction, a decrease in Rb phosphorylation followed by a decrease in total Rb level. These changes were prevented by addition of spermidine. They concluded that polyamine depletion conferred phenotypes resembling cell senescence.

This is in agreement with the observation that polyamine synthesis decreases in senescent cells. Transgenic rodents have been used to study the effect of polyamine pathway modulations on cancer and have yielded a wealth of data, albeit inconclusive and sometime contradictory at the moment. A comprehensive overview of the results obtained in rodents transgenic for polyamine pathway genes has been published [6]. We will here briefly summarize the most important results to illustrate the state of the knowledge between polyamines and cancer. As mentioned earlier, transgenic mice overexpressing human ODC under its own promoter did not exhibit a higher rate of spontaneous tumorigenesis. However, these mice more readily developed skin papillomas in response to a two-stage chemical skin tumorigenesis [52]. In contrast, overexpression of a truncated ODC in the skin caused a higher level of spontaneous skin tumor development [53]. However, transgenic mice overexpressing a dominant-negative form of ODC in the skin showed similar levels of tumor development after induced skin tumorigenesis, casting doubts about the involvement of ODC in tumorigenesis [54]. A further layer of complexity was added using SSAT overexpressing mice, which developed fewer papillomas in the two-stage skin tumorigenesis protocol [55]. When SSAT overexpression was directed to the hair follicle, transgenic animals were more prone to the two-stage skin tumorigenesis [56]. Several transgenic lines of mice overexpressing ODC antizyme in the skin have been shown to develop fewer papillomas in a two-stage skin tumorigenesis [57]. Likewise, the incidence of tumor and their multiplicity in response to N-nitrosomethylbenzylamine in the fore-stomach was decreased in transgenic mice overexpressing ODC antizyme in the basal layer of cell of the fore-stomach epithelium [58].

Although the ability of polyamines to induce cancer is still a matter of debate, it is likely that their high levels in cancer cells can help these cells maintain their proliferative capacity. This could happen by the interaction of polyamines with oncogenes [reviewed in 59]. Polyamines have thus been the target for chemotherapeutic agents for a relatively long time. Such potential chemotherapeutic drugs developed are polyamine synthesis inhibitors, polyamine analogues or polyamine-conjugated compounds. Many reviews and papers have gathered the knowledge on this field [3, 5, 60-64]. The ODC inhibitor DMFO was the first one synthesized and studied. This compound showed no effect during clinical trials, and is currently being studied as a chemopreventive agent rather than a chemotherapeutic one [reviewed in 60]. Another approach is to target not polyamine metabolism itself, but other pathways using polyamines. Transglutaminases catalyze the incorporation of several low molecular weight amines into proteins. Polyamines are substrates for transglutaminases both *in vitro* and *in vivo* [65]. Compounds such as phytochemicals increased transglutaminase activity, and this correlated with less metastatic development of tumors [reviewed in 66]. Other molecules have also been hinted as potential chemopreventive agents. Resveratrol can inhibit polyamine synthesis and activate polyamine catabolism [67]. It has been shown to reduce colitis and thus decrease colon cancer [68]. Ellagic acid depletes the polyamine pool and quercetin leads to inhibition of ODC expression and activity in cell lines [reviewed in 69]. Interestingly, some of these compounds increase life span. Resveratrol can increase life span in invertebrate model organisms although contradictory results have been reported [70, 71, 72 *versus* 73] and it increases life span in mice on a high calorie diet [74] but not when normally fed [75, 76]. Resveratrol could trigger its beneficial effects by modulating S6 kinase activity [77, 78]. Quercetin has been reported to have anti-aging effects in cells [79] but no study in whole organism has been carried out. Rapamycin analogues are currently used as chemotherapeutics [80] and rapamycin also increases life span in several model organisms, including rodents [76, 81-86].

### Mechanisms of action of polyamines (Figure 3)

**Figure 3:**
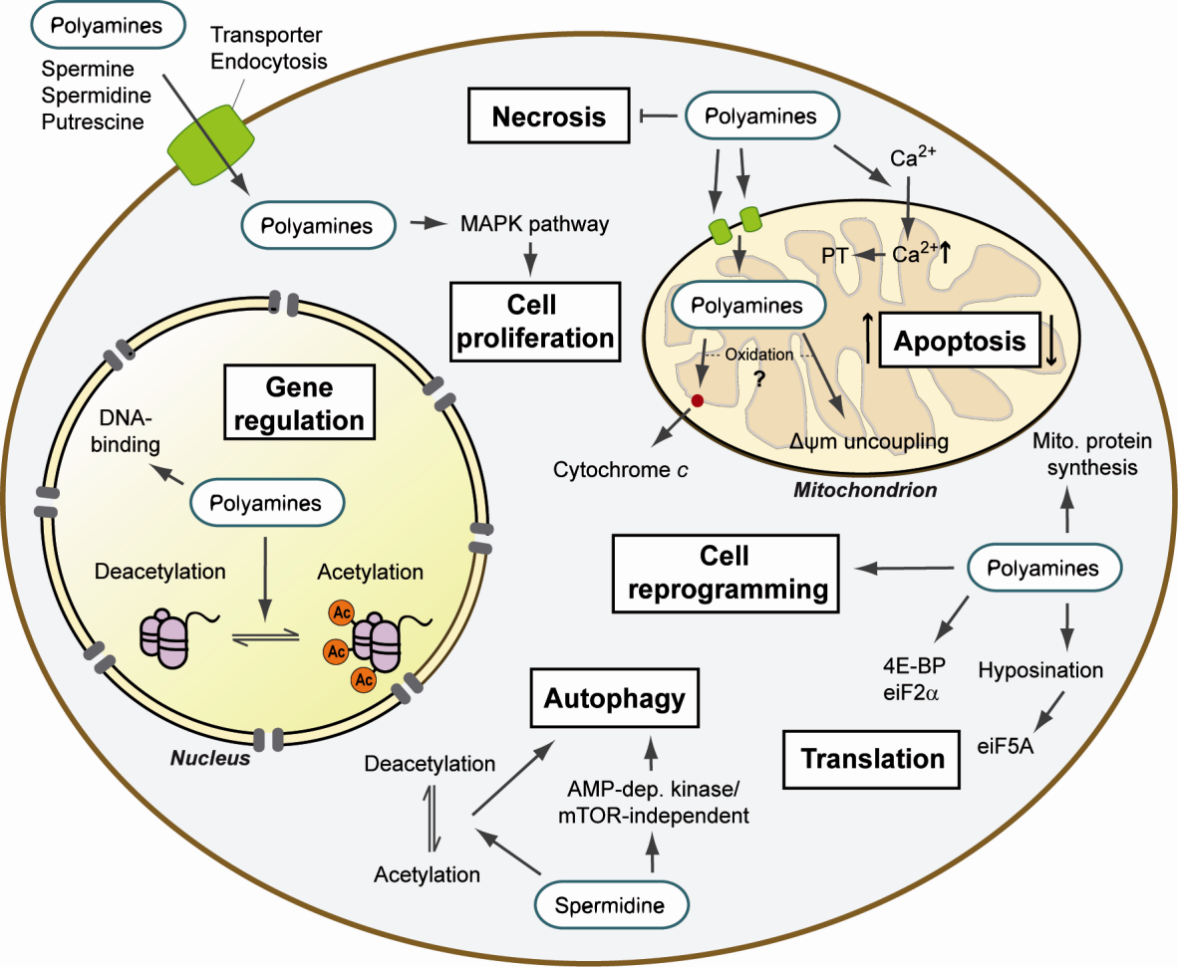
Summary of the cellular mechanisms of action of polyamines Upon entering the cell, polyamines exhibit various functions in the cytoplasm, nucleus and mitochondria. Polyamines are involved in the regulation of cell death and cell proliferation as well as in protein synthesis at the level of both gene expression and translation. Recent evidence also assigned polyamines functions in cell reprogramming and autophagy regulation. Thus, polyamines are involved in a broad array of processes and cellular responses that suggest a complex and important role in the control of cellular life and death. PT: permeability transition; Δψm: mitochondrial membrane potential.

#### Cell growth, apoptosis and necrosis

Polyamines are essential for promoting cell growth. Polyamines could be part of a response of the cells to nutrient availability, especially sugars, that would induce the cells to grow or not [87, 88]. They are also implicated in apoptosis but as often with polyamines, with contradictory results [reviewed in 89]. Polyamines increased Ca^2+^ accumulation in mitochondria, modulating the mitochondrial permeability transition, and triggering apoptosis [90]. Mitochondrial function is very important for life span regulation [91]. Spermine and spermidine interact with the mitochondrial membrane at two specific binding sites, which is the first step of their transport into the mitochondria. Polyamines can also directly promote cytochrome c release, a prelude to apoptosis [92]. However, the effects of polyamines on apoptosis are unclear. High levels of polyamines have been shown to trigger apoptosis in many cell types, but decreased levels can also trigger apoptosis. Furthermore, not polyamines themselves but products derived from polyamine metabolism may be responsible for apoptosis. Spermine decreased the mitochondrial membrane potential and allowed cytochrome c release in a dose-dependent manner in cell free mitochondria preparations [93]. Spermidine and putrescine both also induced uncoupling and cytochrome c release. The authors observed that H_2_O_2_ had the same effect and that catalase decreased the effects of polyamines on mitochondria. Based on these results, the authors suggested that the oxidation products of polyamines, not polyamines themselves, were responsible for uncoupling and cytochrome c release. Finally, polyamines can regulate the other type of cell death, necrosis. We observed that spermidine feeding increased yeast cells chronological life span by reducing necrotic death and that cells unable to synthesize polyamines exhibited higher levels of necrosis [4]. A recent report by Carmona-Gutierrez et al. [94] suggested that the regulation of necrosis by polyamines may work via cathepsin D (CatD). CatD has been involved in cancer cells proliferation and survival [95]. The overexpression of PEP4, the yeast ortholog of CatD, increased chronological life span by reducing necrosis. This anti-necrotic effect was dependent on putrescine and spermidine synthesis, but not on spermine synthesis. Furthermore, PEP4 overexpression prevented the age-related decline of putrescine and spermidine levels.

#### Chromatin structure and gene regulation

Due to their cationic nature, polyamines can strongly bind DNA and change its conformation. Chromatin acetylation plays an important role in the effect of polyamines on DNA [reviewed in 96]. High polyamine levels alter histone acetylation and histone acetylases and deacetylases activities in proliferative cells. For instance, in keratinocytes of transgenic mice overexpressing ODC in the skin, overall acetylation was reduced but both histone acetylases and deacetylases activities were increased [97]. We have recently linked the beneficial effects of spermidine on life span and aging to acetylation [4]. Spermidine reduced acetylation at all studied lysine residues of histone H3 in yeast. We showed that the premature death of *Δspe1* mutants unable to synthesize polyamines was associated with hyperacetylation. In contrast, double *Δiki3/Δsas3* (both known to acetylate histone H3) mutants in yeast showed lower overall acetylation and as expected, were long-lived, produced less reactive oxygen species and showed less necrosis. A screen for the loss of effect of spermidine on life span in yeast showed that the hypoacetylation by spermidine was mainly due to an inhibition of histone acetylases rather than activation of histone deacetylases. Finally, we also observed lower acetylation levels in peripheral blood mononuclear cells. Taken together, the results showed that polyamines affect acetylation, which in turn will affect gene regulation. Corroborating this idea, Handa and Mattoo [98] reported that high levels of spermidine and spermine in transgenic tomato fruit up-regulated the expression of a quarter out of 1066 studied genes and down-regulated half as many compared with wild-type fruits. They studied the effect of each polyamine on the level of 10 chosen proteins. Putrescine had opposite effects on the expression of these proteins from spermine and spermidine, resulting in opposite effects on cellular metabolism in ripening fruits. Spermidine also regulates gene translation. eIF5A regulates translation elongation in yeast, *Drosophila* and mammalian cells [99]. It is the only protein containing the amino acid residue hypusine. eIF5A-hypusine is specifically synthesized from spermidine by the enzyme deoxyhypusine hydroxylase via the formation of eIF5A-deoxyhypusine by deoxyhypusine synthase (DHS, Figure 2). This reaction and the formation of eIF5A are important for cell growth as GC7, an inhibitor of DHS, inhibits cell proliferation. However, GC7 also leads to a reduction of polyamines, which could lead to loss of cell proliferation too. It was thought that hypusination may have been the major requirement of polyamines for cell proliferation. However, polyamines do not act on cell growth only via hypusination, and also regulate translation initiation via eIF2α and 4E-BP. In fact, spermine was involved in the initiation of mitochondrial protein synthesis by increasing the binding of fMet-tRNA to the 28S small ribosomal subunit [100].

It thus seems that polyamines, by modulating acetylation levels and protein synthesis, will trigger varied changes that can potentially lead to complex cellular responses. Such actions of polyamines may help explain how they can promote both cell growth and cell death and can show such a complex involvement in aging, stress and diseases. Finally, it has recently been reported that spermidine was also able to enhance the induced reprogramming of SKOM (Sox2, Klf4, Oct4 and c-Myc)-infected primary mouse embryonic fibroblasts into pluripotent stem cells [101].

#### Autophagy

Autophagy is the mechanism responsible for the degradation of intracellular macromolecules and organelles. Autophagy is an essential function in development and survival as it will dispose of unwanted molecules such as damaged molecules during stress, aging or diseases, or during developmental remodeling. Autophagy is involved in many diseases [102-104]. Many genetic and non genetic manipulations increasing life span induce autophagy [105] and autophagy appears as a point of convergence of many genetic pathways involved in aging [106]. We have shown that induction of autophagy was probably the main mechanism of action of spermidine to trigger its beneficial effects on life span and aging [4, 94, 107, 108]. First, we found that autophagy-related genes in yeast were up-regulated by spermidine addition. We also measured that the Atg7 promoter was hyperacetylated with spermidine compared with the promoters of neighboring genes and despite the overall hypoacetylation effect of spermidine. We then confirmed that spermidine induced autophagy in yeast, worms, flies and human cells by direct measurements of autophagy levels. Finally, we showed that life extension by spermidine was abrogated in yeast, worms and flies autophagy mutants. Morselli et al. [109] investigated by which mechanisms spermidine and resveratrol (both increasing life span) induced autophagy. Both compounds induced autophagy in human colon cancer cells. However, decreased SIRT1 activity by siRNA or SIRT1 inhibitor abrogated resveratrol-induced but not spermidine-induced autophagy in these cells. Spermidine induced autophagy, increased survival and decreased ROS production with age in both wild-type and Δ*sir2* mutant yeast. In worms, spermidine-induced autophagy was slightly decreased in *sir1.2* mutants but spermidine had the same effect on life span in both wild-type and mutants. It appeared that the two compounds induced autophagy by distinct pathways. However, they both had the same effect on protein phosphorylation, suggesting that the pathways converge. The two compounds induced autophagy through AMP-dependent kinase/mTOR-independent convergent pathways. The authors then studied the relation between acetylation and induction of autophagy. Spermidine and resveratrol induced acetylation changes in 375 proteins, 170 of which belong to the human autophagy protein network. They found that in the cytoplasm, the compounds induced more convergent deacetylation than acetylation. The reverse was observed in the nucleus. By using cytoplasts (enucleated cells), the authors could show that short-term autophagy was regulated by cytoplasmic (de)acetylation reactions. These results confirmed that autophagy is an important mechanism of action of spermidine. It would be now interesting to know whether the other polyamines can also induce autophagy and study further the relationship between autophagy, aging, and diseases.

#### Signaling pathways

Polyamines interact with signaling pathways to modulate cellular response. CK2 is a kinase ubiquitously expressed with important roles in cell proliferation and survival [e.g., 110]. Stark et al. [111] investigated whether CK2 was a target of polyamines by which they would modulate the MAPK pathway (Ras, Raf, MERK, Erk pathway). It had already been shown that polyamines regulate CK2 activity *in vitro* [112]. By using Drosophila S2 cell cultures, Stark et al. [111] showed that CK2 formed a complex with KSR (kinase suppressor of Ras) and phosphorylated Draf. Spermine decreased Draf phosphorylation by CK2 *in vitro*. In S2 cells, spermine decreased Erk activation (last step in the pathway) but putrescine and spermidine only had weak effects. In contrast, the concomitant addition of putrescine or spermidine increased Erk activation. The authors suggested that CK2 could sense relative polyamine levels and translate the information to the MAPK pathway to trigger the appropriate cellular response.

## CONCLUSIONS AND PERSPECTIVES

In this review, we have seen how complex the interaction of polyamines with aging and diseases is. So far, polyamines have been reported to be beneficial for aging. They can increase life span and delay kidney glomerular atrophy. The various studies have also shown mainly positive effects of polyamines on stress resistance. In plants, exogenous spermine treatment and increased polyamine synthesis by genetic means lead to higher stress resistance. Spermidine supplementation increases stress resistance and decreases age-related oxidative stress markers in several model organisms. High levels of polyamines are associated with many diseases, including cancers. In these cases, polyamines have mostly been thought of as exacerbating, if not causal, factors. However, there has been no firm evidence that modulation of polyamine levels in itself can cause diseases, whether cancer or other age-related diseases such as Parkinson's disease. Furthermore, transgenic models with increased polyamine synthesis or catabolism have been shown to exhibit both higher and lower levels of tumorigenesis after chemically-induced tumorigenesis protocols. Bringing together the results of the various fields of research on the effects and roles of polyamines has drawn a complex picture and we think it should now be impossible to study polyamines in one field without taking into account the knowledge gathered in the others. One of the first complexities is that polyamines are usually taken as a family of similar molecules. However, as described, different polyamines can have different, even opposite effects. It is thus urgent to understand the role of each polyamine as well as of their combination (a natural occurrence in organisms) on aging and diseases. This is especially important for aging, as for the moment, only spermidine alone or a diet low or rich in polyamines in general have been studied for their effects on life span and aging. We also need to further our understanding of the current knowledge. For instance, the effect of polyamines on life span and aging in rodents should be investigated as the first study showed promising, but so far not definitive results. Finally, the work relating polyamines and aging have mainly focused on life span and the next step should investigate what are the effects of polyamines on aging and whether they improve quality of life in older organisms.

It is becoming urgent to resolve the involvement of polyamines in disease. The hypothesis that they could represent an early compensatory mechanism should be given attention. It is however an arduous task, as if this mechanism is efficient, the disease will not progress and the effect of polyamines will thus not be easily identifiable. The disease may be seen when the compensatory mechanism has failed and thus the disease can progress, but the compensatory mechanism by polyamines is maintained, hence the apparent involvement of polyamines in the disease.

Tools that could improve our understanding of the roles of polyamines are models exhibiting an accumulation of spermidine or spermine. To date, the transgenic models available, mainly targeting ODC or SSAT, lead to an accumulation of putrescine, with or without a concomitant depletion of spermidine and spermine. Recently, the description of mice overexpressing spermidine synthase was reported [113]. These mice exhibited high levels of spermidine in heart, muscle and liver tissue. They were viable and fertile and it would be interesting now to use this new model in the study of the effect of spermidine on aging, stress and diseases. It is also important to understand the proximal effects on polyamine metabolism of exogenous addition of a single polyamine. For instance, we have observed that flies fed spermidine exhibited higher levels of spermidine, but also of putrescine [4], suggesting an adaptation of polyamine metabolism to the influx of one polyamine.

To conclude, our understanding of the role of polyamines on aging and diseases has now reached a level where it seems that polyamines (particularly spermidine) can be beneficial for aging in healthy organisms but that they may be harmful when disease appears. So far, most of the interventions known to beneficial to aging (genetic interventions, dietary restriction, rapamycin) have also been reported to be helpful against age-related diseases [see 114 and 115 for a discussion] and it would be an unusual characteristic of polyamines to exhibit such a dual effect on aging and diseases. Bringing together the various fields of research interested in the roles of polyamines will help us to further unravel the effects of polyamines to a point where the implementation of their use in humans would be the next step.

## SUPPLEMENTAL DATA



## References

[R1] Bachrach U (2010). The early history of polyamine research. Plant Physiol Biochem.

[R2] Hussain SS, Ali M, Ahmad M, Siddique KHM (2011). Polyamines: Natural and engineered abiotic and biotic stress tolerance in plants. Biotechnol Adv.

[R3] Wallace HM, Fraser AV (2004). Inhibitors of polyamine metabolism: Review article. Amino acids.

[R4] Eisenberg T, Knauer H, Schauer A, Fussi H, Büttner S, Carmona-Gutierrez D, Ruckenstuhl C, Fahrenkrog B, Deszcz L, Hartl R, Weiskopf D, Grubeck-Loebenstein B, Herker E (2009). Induction of autophagy by spermidine promotes longevity. Nature Cell Biol.

[R5] Wallace HM, Fraser AV, Hughes A (2003). A perspective of polyamine metabolism. Biochem J.

[R6] Jänne J, Alhonen L, Pietilä M, Keinänen TA (2004). Genetic approaches to the cellular functions of polyamines in mammals. Eur J Biochem.

[R7] Igarashi K, Kashiwagi K (2010). Characteristics of cellular polyamine transport in prokaryotes and eukaryotes. Plant Physiol Biochem.

[R8] Perez-Leal O, Merali S (2011). Regulation of polyamine metabolism by translational control. Amino acids.

[R9] Higashi K, Ishigure H, Demizu R, Uemura T, Nishino K, Yamaguchi A, Kashiwagi K, Igarashi K (2008). Identification of a spermidine excretion protein complex (MdtJI) in Escherichia coli. J Bacteriol.

[R10] Teixeira MC, Cabrito TR, Hanif ZM, Vargas RC, Tenreiro S, Sá-Correia I (2010). Yeast response and tolerance to polyamine toxicity involving the drug:H^+^ antiporter Qdr3 and the transcription factors Yap1 and Gcn4. Microbiol.

[R11] Fukuchi J, Hiipakka RA, Kokonitis JM, Nishimura K, Igarashi K, Liao S (2004). TATA-binding protein-associated factor 7 regulates polyamine transport activity and polyamine-analog-induced apoptosis. J Biol Chem.

[R12] Uemura T, Yerushalmi HF, Tsaprailis G, Stringer DE, Pastorian KE, Hawel L, Byus CV, Gerner EW (2008). Identification and characterization of a diamine exporter in colon epithelial cells. J Biol Chem.

[R13] Poulin R, Casero RA, Soulet D (2011). Recent advances in the molecular biology of metazoan polyamine transport. Amino acids.

[R14] Scalabrino G, Ferioli ME (1984). Polyamines in mammalian ageing: an oncological problem, too? A review. Mech Ageing Dev.

[R15] Nishimura K, Shiina R, Kashiwagi K, Iagarashi K (2006). Decrease in polyamines with aging and their ingestion from food and drink. J Biochem.

[R16] Vivó M, de Vera N, Cortés R, Mengod G, Camón L, Martínez E (2001). Polyamines in the basal ganglia of human brain. Influence of aging and degenerative movement disorders. Neurosci Lett.

[R17] Liu P, Gupta N, Jing Y, Zhang H (2008). Age-related changes in polyamines in memory-associated brain structures in rats. Neurosci.

[R18] Fraga MF, Berdasco M, Borja Diego L, Rodríguez R, Cañal MJ (2004). Changes in polyamine concentration associated with aging in Pinus radiata and Prunus persica. Tree Physiol.

[R19] Serafini-Fracassini D, Del Luca S, Monti F, Poli F, Sacchetti G, Bregoli AM, Biondi S, Della Mea M (2002). Transglutaminase activity during senescence and programmed cell death in the corolla of tobacco (Nicotiana tabacum) flowers. Cell Death Diff.

[R20] Serafini-Fracassini D, Di Sandro A, Del Luca S (2010). Spermine delays leaf senescence in Lactuca sativa and prevents the decay of chloroplast photosystems. Plant Physiol Biochem.

[R21] Suppola S, Heikkinen S, Parkkinen JJ, Uusi-Oukari M, Korhonen VP, Keinänen T, Alhonen L, Jänne J (2001). Concurrent overexpression of ornithine decarboxylase and spermidine/spermine N^1^-acetyltransferase further accelerates the catabolism of hepatic polyamines in transgenic mice. Biochem J.

[R22] Cerrada-Gimenez M, Pietilä M, Loimas S, Pirinen E, Hyvönen MT, Keinänen TA, Jänne J, Alhonen L (2011). Continuous oxidative stress due to activation of polyamine catabolism accelerates aging and protects against hepatotoxic insults. Transgenic Res.

[R23] Soda K, Kano Y, Sakuragi M, Takao K, Lefor A, Konishi F (2009). Long-term oral polyamine intake increases blood polyamine concentrations. J Nutr Sci Vitaminol.

[R24] Soda K, Dobashi Y, Kano Y, Tsujinaka S, Konishi F (2009). Polyamine-rich food decreases age-associated pathology and mortality in aged mice. Exp Gerontol.

[R25] Alcázar R, Cuevas JC, Planas J, Zarza X, Bortolotti C, Carrasco P, Salinas J, Tiburcio AF, Altabella T (2011). Integration of polaymines in the cold acclimation response. Plant Sci.

[R26] Mohapatra S, Cherry S, Minocha R, Majumdar R, Thangavel P, Long S, Minocha SC (2010). The response of high and low polyamine-inducing cell lines to aluminum and calcium stress. Plant Physiol Biochem.

[R27] Wang J, Sun PP, Chen CL, Wang Y, Fu XZ, Liu JH (2011). An arginine decarboxylase gene PtADC from Poncirus trifoliate confers abiotic stress tolerance and promotes primary root growth in Arabidopsis. J Exp Bot.

[R28] Gonzalez ME, Marco F, Gómez Minguet E, Carrasco-Sorli P, Blázquez MA, Carbonell J, Ruiz OA, Pieckenstain FL (2011). Perturbation of spermine synthase gene expression and transcript profiling provide new insights on the role of the tetraamine spermine in Arabidopsis thaliana defense against Pseudomonas viridiflava. Plant Physiol.

[R29] Kaasinen SK, Oksman M, Alhonen L, Tanila H, Jänne J (2004). Spermidine/spermine N^1^-acetyltransferase overexpression in mice induces hypoactivity and spatial learning impairment. Pharmacol Biochem Behav.

[R30] Vigne P, Frelin C (2008). The role of polyamines in protein-dependent hypoxic tolerance of Drosophila. BMC Physiol.

[R31] Gomes-Trolin C, Nygren I, Aquilonius SM, Askmark H (2002). Increased red blood cell polyamines in ALS and Parkinson's disease. Exp Neurol.

[R32] Yatin SM, Yatin M, Aulick T, Ain KB, Butterfield DA (1999). Alzheimer's amyloid β-peptide associated free radicals increase rat embryonic neuronal polyamine uptake and ornithine decarboxylase activity: protective effect of vitamin E. Neurosci Lett.

[R33] Antony T, Hoyer W, Cherny D, Heim G, Jovin TM, Subramaniam V (2003). Cellular polyamines promote the aggregation of α-synuclein. J Biol Chem.

[R34] Grabenauer M, Bernstein SL, Lee JC, Wyttenbach T, Dupuis NF, Gray HB, Winkler JR, Bowers MT (2008). Spermine binding to Parkinson's protein α-synuclein and its disease-related A30P and A53T mutants. J Phys Chem B.

[R35] Lewandowski NM, Ju S, Verbitsky M, Ross B, Geddie ML, Rockenstein E, Adama A, Muhammad A, Vonsattel JP, Ringe D, Cote L, Lindquist S, Masliah E (2010). (2010) Polyamine pathway contributes to the pathogenesis of Parkinson disease. Proc Natl Acad Sci USA.

[R36] Duan B, Wang YZ, Yang T, Chu XP, Yu Y, Huang Y, Cao H, Hansen J, Simon RP, Zhu MX, Xiong ZG, Xu TL (2011). Extracellular spermine exacerbates ischemic neuronal injury through sensitization of ASIC1a channels to extracellular acidosis. J Neurosci.

[R37] Bell MR, Belarde JA, Johnson HF, Aizenman CD (2011). A neuroprotective role for polyamines in a Xenopus tadpole model of epilepsy. Nature Neurosci.

[R38] Jung YS, Kim SJ, Kwon DY, Kim YC (2011). Metabolomic analysis of sulphur-containing substances and polyamines in regenerating rat liver. Amino Acids.

[R39] Puntambekar SS, Davis DS, Hawel L, Crane J, Byus CV, Carson MJ (2011). Lps-induced Ccl2 expression and macrophage influx into the murine central nervous system is polyamine-dependent. Brain Behav Immun.

[R40] Soda K (2010). Polyamine intake, dietary pattern, and cardiovascular disease. Med Hypotheses.

[R41] Lentini A, Tabolacci C, Mattioli P, Provenzano B, Beninati S (2011). Spermidine delays eye lens opacification in vitro by suppressing transglutaminase-catalyzed crystalline cross-linking. Protein J.

[R42] Guo X, Harada C, Namekata K, Kimura A, Mitamura Y, Yoshida H, Matsumoto Y, Harada T (2011). Spermidine alleviates severity of murine experimental autoimmune encephalomyelitis. Inves Ophtalmol Vis Sci.

[R43] Nishimura K, Yanase T, Nakagawa H, Matsuo S, Ohnishi Y, Yamasaki S (2009). Effect of polyamine-deficient chow on Trypanosoma brucei brucei infection in rats. J Parasitol.

[R44] Reyes-Becerril M, Ascencio-Valle F, Tovar-Ramírez D, Meseguer J, Esteban MA (2010). Effects of polyamines on cellular innate immune response and the expression of immune-relevant genes in gilthead seabream leucocytes. Fish Shellfish Immunol.

[R45] Uehara Y, Takahashi Y, Berberich T, Miyazaki A, Takahashi H, Matsui K, Ohme-Takagi M, Saitoh H, Terauchi R, Kusano T (2005). Tobacco ZFT1, a transcriptional repressor with a Cys2/His2 type zinc finger motif that functions in spermine-signaling pathway. Plant Mol Biol.

[R46] Shah P, Nanduri B, Swiatlo E, Ma Y, Pendarvis K (2011). Polyamine biosynthesis and transport mechanisms are crucial for fitness and pathogenesis of Streptococcus pneumonia. Microbiol.

[R47] Hibshoosh H, Johnson M, Weinstein IB (1991). Effects of overexpression of ornithine decarboxylase (ODC) on growth control and oncogene-induced cell transformation. Oncogene.

[R48] Auvinen M, Paasinen A, Andersson LC, Hölttä E (1992). Ornithine decarboxylase activity is critical for cell transformation. Nature.

[R49] Alhonen L, Halmekytö M, Kosma VM, Wahlfors J, Kauppinen R, Jänne J (1995). Life-long over-expression of ornithine decarboxylase (ODC) gene in transgenic mice does not lead to generally enhanced tumorigenesis or neuronal degeneration. Int J Cancer.

[R50] Bernacki RJ, Oberman EJ, Sewerymiak KE, Atwood A, Bergeron RJ, Porter CW (1995). Preclinical antitumor efficacy of the polyamine analogue N^1^, N^11^-diethylnorspermine administered by multiple injection or continuous infusion. Clin Cancer Res.

[R51] Kramer DL, Chang BD, Chen Y, Diegelman P, Alm K, Black AR, Roninson IB, Porter CW (2001). Polyamine depletion in human melanoma cells leads to G1 arrest associated with induction of p21^WAF1/CIP1/SDI1^, changes in the expression of p21-regulated genes, and a senescence-like phenotype. Cancer Res.

[R52] Halmekytö M, Syrjänen K, Jänne J, Alhonen L (1992). Enhanced papilloma formation in response to skin tumor promotion in transgenic mice overexpressing the human ornithine decarboxylase gene. Biochem Biophys Res Commun.

[R53] Megosh L, Gilmour SK, Rosson D, Soler AP, Blessing M, Sawicki JA, O'Brien TG (1995). Increased frequency of spontaneous skin tumors in transgenic mice which overexpress ornithine decarboxylase. Cancer Res.

[R54] Shantz LM, Guo Y, Sawicki JA, Pegg AE, O'Brien TG (2002). Overexpression of a dominant-negative ornithine decarboxylase in mouse skin: effect on enzyme activity and papilloma formation. Carcinogenesis.

[R55] Pietilä M, Parkkinen JJ, Alhonen L, Jänne J (2001). Relation of skin polyamines to the hairless phenotype in transgenic mice overexpressing spermidine/spermine N^1^-acetyltransferase. J Invest Dermatol.

[R56] Coleman CS, Pegg AE, Megosh LC, Guo Y, Sawicki JA, O'Brien TG (2002). Targeted expression of spermidine/spermine N^1^-acetyltransferase increases susceptibility to chemically induced skin carcinogenesis. Carcinogenesis.

[R57] Feith DJ, Shantz LM, Pegg AE (2001). Targeted antizyme expression in the skin of transgenic mice reduces tumor promoter induction of ornithine decarboxylase and decreases sensitivity to chemical carcinogenesis. Cancer Res.

[R58] Fong LY, Feith DJ, Pegg AE (2003). Antizyme overexpression in transgenic mice reduces cell proliferation, increases apoptosis, and reduces N-nitrosomethylbenzylamine-induced forestomach carcinogenesis. Cancer Res.

[R59] Gerner EW, Meyskens FL (2004). Polyamines and cancer: old molecules, new understanding. Nat Rev Cancer.

[R60] Casero RA, Marton LJ (2007). Targeting polyamine metabolism and function in cancer and other hyperproliferative diseases. Nat Rev Drug Discov.

[R61] Amendola R, Cervelli M, Fratini E, Polticelli F, Sallustio DE, Mariottini P (2009). Spermine metabolism and anticancer therapy. Curr Cancer Drug Targets.

[R62] Senanayake MDT, Amunugama H, Boncher TD, Casero RA, Woster PM (2009). Design of polyamine-based therapeutic agents: new targets and new directions. Essays Biochem.

[R63] Szumilak M, Szulawska-Mroczek A, Koprowska K, Stasiak M, Lewgowd W, Stanczak A, Czyz M (2010). Synthesis and in vitro biological evaluation of new polyamine conjugates as potential anticancer drugs. Eur J Med Chem.

[R64] Babbar N, Gerner EW, Senn HJ, Otto F (2011). Targeting polyamines and inflammation for cancer prevention. Clinical Cancer Prevention.

[R65] Beninati S, Piacentini M, Cocuzzi ET, Autuori F, Folk JE (1988). Polyamines as physiological substrates for transglutaminases. Biochim Biophys Acta.

[R66] Lentini A, Tabolacci C, Provenzano B, Rossi S, Beninati S (2010). Phytochemicals and protein – polyamine conjugates by transglutaminase as chemopreventive and chemotherapeutic tools in cancer. Plant Physiol Biochem.

[R67] Wolter F, Ulrich S, Stein J (2004). Molecular mechanisms of the chemopreventive effects of resveratrol and its analogs in colorectal cancer: key role of polyamine?. J Nutr.

[R68] Hofseth LJ, Singh UP, Singh NP, Nagarkatti M, Nagarkatti PS (2010). Taming the beast within: resveratrol suppresses colitis and prevents colon cancer. Aging.

[R69] Saunders FR, Wallace HM (2010). On the natural chemoprevention of cancer. Plant Physiol. Biochem.

[R70] Parashar V, Rogina B (2009). dSir2 mediates the increased spontaneous physical activity in flies on calorie restriction. Aging.

[R71] Bauer JH, Morris SN, Chang C, Flatt T, Wood JG, Helfand SL (2009). dSir2 and Dmp53 interact to mediate aspects of CR-dependent lifespan extension in D. melanogaster. Aging.

[R72] Wood JG, Rogina B, Lavu S, Howitz K, Helfand SL, Tatar M, Sinclair D (2004). Sirtuin activators mimic caloric restriction and delay ageing in metazoans. Nature.

[R73] Bass TM, Weinkove D, Houthoofd K, Gems D, Partridge L (2007). Effects of resveratrol on lifespan in Drosophila melanogaster and Caenorhabditis elegans. Mech Ageing Dev.

[R74] Baur JA, Pearson KJ, Price NL, Jamieson HA, Lerin C, Kalra A, Prabhu VV, Allard JS, Lopez-Lluch G, Lewis K, Pistell PJ, Poosala S, Becker KG (2006). Resveratrol improves health and survival of mice on a high-calorie diet. Nature.

[R75] Pearson KJ, Baur JA, Lewis KN, Peshkin L, Price NL, Labinskyy N, Swindell WR, Kamara D, Minor RK, Perez E, Jamieson HA, Zhang Y, Dunn SR (2008). Resveratrol delays age-related deterioration and mimics transcriptional aspects of dietary restriction without extending life span. Cell Metab.

[R76] Miller RA, Harrison DE, Astle CM, Baur JA, Boyd AR, de Cabo R, Fernandez E, Flurkey K, Javors MA, Nelson JF, Orihuela CJ, Pletcher S, Sharp ZD (2010). Rapamycin, but not resveratrol or simvastatin, extends lifespan of genetically heterogeneous mice. J Gerontol A Biol Sci Med Sci.

[R77] Armour SM, Baur JA, Hsieh SN, Land-Bracha A, Thomas SM, Sinclair DA (2009). Inhibition of mammalian S6 kinase by resveratrol suppresses autophagy. Aging.

[R78] Blagosklonny MV (2009). Inhibition of S6K by resveratrol: in search of the purpose. Aging.

[R79] Chondrogianni N, Kapeta S, Chinou I, Vassilatou K, Papassideri I, Gonos ES (2010). Anti-ageing and rejuvenating effects of quercetin. Exp Gerontol.

[R80] Martelli AM, Evangelisti C, Chiarini F, McCubrey JA (2010). The phosphatidylinositol 3-kinase/Akt/mTOR signaling network as a therapeutic target in acute myelogenous leukemia patients. Oncotarget.

[R81] Powers RW, Kaeberlein M, Caldwell SD, Kennedy BK, Fields S (2006). Extension of chronological lifespan in yeast by decreased TOR pathway signaling. Genes Dev.

[R82] Alvers AL, Wood MS, Hu D, Kaywell AC, Dunn WA, Aris JP (2009). Autophagy is required for extension of yeast chronological life span by rapamycin. Autophagy.

[R83] Bjedov I, Toivonen JM, Kerr F, Slack C, Jacobson J, Foley A, Partridge L (2010). Mechanisms of lifespan extension by rapamycin in the fruit fly Drosophila melanogaster. Cell Metab.

[R84] Harrison DE, Strong R, Sharp ZD, Nelson JF, Astle CM, Flurkey K, Nadon NL, Wilkinson JE, Frenkel K, Carter CS, Pahor M, Javors MA, Fernandez E (2009). Rapamycin fed late in life extends lifespan in genetically heterogeneous mice. Nature.

[R85] Pan Y, Shadel GS (2009). Extension of chronological life span by reduced TOR signaling requires down-regulation of Sch9p and involves increased mitochondrial OXPHOS complex density. Aging.

[R86] Demidenko ZN, Zubova SG, Bukreeva EI, Pospelov VA, Pospelova TV, Blagosklonny MV (2009). Rapamycin decelerates cellular senescence. Cell Cycle.

[R87] Ruckenstuhl C, Carmona-Gutierrez D, Madeo F (2010). The sweet taste of death: glucose triggers apoptosis during yeast chronological aging. Aging.

[R88] Ruckenstuhl C, Büttner S, Carmona-Gutierrez D, Eisenberg T, Kroemer G, Sigrist SJ, Fröhlich KU, Madeo F (2009). The Warburg effect suppresses oxidative stress induced apoptosis in a yeast model for cancer. PLoS One.

[R89] Bjelaković G, Stojanović I, Jevtović Stoimenov T, Pavlović D, Kocić G, Rossi S, Tabolacci C, Nikolić J, Sokolović D, Bjelakovic LJ (2010). Metabolic correlations of glucocorticoids and polyamines in inflammation and apoptosis. Amino Acids.

[R90] Salvi M, Toninello A (2004). Effects of polyamines on mitochondrial Ca(2+) transport. Biochim Biophys Acta.

[R91] Heeren G, Rinnerthaler M, Laun P, von Seyerl P, Kössler S, Klinger H, Hager M, Bogengruber E, Jarolim S, Simon-Nobbe B, Schüller C, Carmona-Gutierrez D, Breitenbach-Koller L (2009). The mitochondrial ribosomal protein of the large subunit, Afo1p, determines cellular longevity through mitochondrial back-signaling via TOR1. Aging.

[R92] Stefanelli C, Stanic' I, Zini M, Bonavita F, Flamigni F, Zambonin L, Landi L, Pignatti C, Guarnieri C, Caldarera CM (2000). Polyamines directly induce release of cytochrome c from heart mitochondria. Biochem J.

[R93] Maccarrone M, Bari M, Battista N, Di Rienzo M, Falciglia K, Finazzi Agrò A (2001). Oxidation products of polyamines induce mitochondrial uncoupling and cytochrome c release. FEBS Lett.

[R94] Carmona-Gutierrez D, Bauer MA, Ring J, Knauer H, Eisenberg T, Büttner S, Ruckenstuhl C, Reisenbichler A, Magnes C, Rechberger GN, Birner-Gruenberger R, Jungwirth H, Fröhlich KU (2011). The propeptide of yeast cathepsin D inhibits programmed necrosis. Cell Death Dis.

[R95] Liaudet-Coopman E, Beaujoin M, Derocq D, Garcia M, Glondu-Lassis M, Laurent-Matha V, Prébois C, Rochefort H, Vignon F (2006). Cathepsin D: newly discovered functions of a long-standing aspartic protease in cancer and apoptosis. Cancer Lett.

[R96] Hobbs CA, Gilmour SK, Wang JY, Casero RA (2006). Role of polyamines in the regulation of chromatin acetylation. Polyamine Cell Signalling: Physiology, Pharmacology, and Cancer Research.

[R97] Hobbs C. A., Paul B. A., Gilmour S. K. (2002). Deregulation of polyamine biosynthesis alters intrinsic histone acetyltransferase and deacetylase activities in murine skin and tumors. Cancer Res.

[R98] Handa AK, Mattoo AK (2010). Differential and functional interactions emphasize the multiple roles of polyamines in plants. Plant Physiol Biochem.

[R99] Landau G, Bercovich Z, Park MH, Kahana C (2010). The role of polyamines in supporting growth of mammalian cells is mediated through their requirement for translation initiation and elongation. J Biol Chem.

[R100] Christian BE, Haque ME, Spremulli LL (2010). The effect of spermine on the initiation of mitochondrial protein synthesis. Biochem Biophys Res Comm.

[R101] Chen T, Shen L, Yu J, Wan H, Guo A, Chen J, Long Y, Zhao J, Pei G (2011). Rapamycin and other longevity-promoting compounds enhance the generation of mouse induced pluripotent stem cells. Aging Cell.

[R102] Levine B, Kroemer G (2008). Autophagy in the pathogenesis of disease. Cell.

[R103] Mizushima N, Levine B, Cuervo AM, Klionsky DJ (2008). Autophagy fights disease through cellular self-digestion. Nature.

[R104] Fleming A, Noda T, Yoshimori T, Rubinsztein DC (2011). Chemical modulators of autophagy as biological probes and potential therapeutics. Nat Chem Biol.

[R105] Madeo F, Tavernarakis N, Kroemer G (2010). Can autophagy promote longevity?. Nat Cell Biol.

[R106] Markaki M, Tavernarakis N (2011). The role of autophagy in genetic pathways influencing ageing. Biogerontol.

[R107] Morselli E, Galluzzi L, Kepp O, Criollo A, Maiuri MC, Tavernarakis N, Madeo F, Kroemer G (2009). Autophagy mediates pharmacological lifespan extension by spermidine and resveratrol. Aging.

[R108] Madeo F, Eisenberg T, Büttner S, Ruckenstuhl C, Kroemer G (2010). Spermidine: a novel autophagy inducer and longevity elixir. Autophagy.

[R109] Morselli E, Mariño G, Bennetzen MV, Eisenberg T, Megalou E, Schroeder S, Cabrera S, Bénit P, Rustin P, Criollo A, Kepp O, Galluzzi L, Shen S (2011). Spermidine and resveratrol induce autophagy by distinct pathways converging on the acetylproteome. J Cell Biol.

[R110] St-Denis NA, Litchfield DW (2009). From birth to death: the role of protein kinase CK2 in the regulation of cell proliferation and survival. Cell Mol Life Sci.

[R111] Stark F, Pfannstiel J, Klaiber I, Raabe T (2011). Protein kinase CK2 links polyamine metabolism to MAPK signaling in Drosophila. Cell Signal.

[R112] Niefind K, Raaf J, Issinger OG (2009). Protein kinase CK2 in health and disease: Protein kinase CK2: from structures to insights. Cell Mol Life Sci.

[R113] Shi C, Welsh PA, Sass-Kuhm S, Wang X, McCloskey DE, Pegg AE, Feith DJ (2011). Characterization of transgenic mice with overexpression of spermidine synthase. Amino acids.

[R114] Sharp ZD, Richardson A (2011). Aging and cancer: can mTOR inhibitors kill two birds with one drug?. Targ Oncol.

[R115] Blagosklonny MV (2010). Rapamycin and quasi-programmed aging: four years later. Cell Cycle.

